# Progressive Ankle Subluxation Following Panfibular Osteomyelitis Requiring Fibular Resection

**DOI:** 10.7759/cureus.24112

**Published:** 2022-04-13

**Authors:** Neeraj Vij, Ashish S Ranade, Mohan V Belthur

**Affiliations:** 1 Department of Orthopedic Surgery, University of Arizona College of Medicine - Phoenix, Phoenix, USA; 2 Blooming Buds Centre for Pediatric Orthopaedics, Deenanath Mangeshkar Hospital and Research Centre, Pune, IND; 3 Herbert J. Louis Center for Pediatric Orthopedics, Phoenix Children's Hospital, University of Arizona College of Medicine - Phoenix, Phoenix, USA

**Keywords:** pediatric foot deformity, superolateral talar subluxation, tibiotalar arthrodesis, sepsis, staphylococcal osteomyelitis

## Abstract

A 10-month-old boy presented with fever, a swollen left leg, and septicemic shock. He was diagnosed with panfibular osteomyelitis. Failure of combined medical and surgical treatment to achieve source control necessitated fibular resection. He subsequently developed a progressive superolateral subluxation of his left ankle, valgus deformity, and brace intolerance. Tibiotalar arthrodesis resulted in a stable plantigrade ankle, excellent weight-bearing ability, and a minor leg-length discrepancy at the 14-month postoperative follow-up.

## Introduction

Fibular osteomyelitis is uncommon and the literature is limited to case reports [1-4] and case series [5-6]. The involvement is usually restricted to the proximal, middle, or distal fibula. Panfibular osteomyelitis has very rarely been reported in the literature [7]. Staphylococcus aureus is the most commonly reported organism [5] though there are reports of anaerobic, meningococcal, fungal, and mycobacterial cases [4,8-10]. Medical management alone is often successful [2,4] and generally consists of four to six weeks of antibiotics [2,4]. Indications for surgical treatment include the presence of a subperiosteal/intraosseous abscess or a sequestrum.

The surgical treatment of pandiaphyseal osteomyelitis is also well described by sparing five centimeters of the fibula proximally and distally [7]. Distraction osteogenesis has shown good promise in reconstructing fibula loss in the setting of osteomyelitis [11]. The outcome of localized osteomyelitis of the fibula is generally favorable [5]. However, the surgical management of panfibular osteomyelitis is not well-described.

The aim of this study is to report the outcome of fibular resection for panfibular osteomyelitis and present tibiotalar arthrodesis as a salvage option for progressive, painful ankle subluxation in the setting of fibular resection.

IRB and informed consent

Our institutional review board (IRB) approved this case report (IRB No. 21-214). The patient and his parents were informed that data concerning the case would be submitted for publication and they provided consent.

## Case presentation

Initial presentation

A 10-month-old boy presented with fever, a swollen left leg, and septicemic shock to the emergency department. Clinical evaluation was consistent with compartment syndrome of the left leg and multiple organ dysfunction (MODS). He was admitted to the pediatric intensive care unit and started on intravenous vancomycin, ceftriaxone, and clindamycin and resuscitated for multiple organ failure. A double-incision fasciotomy of the left leg and drainage of the subperiosteal abscess around the fibula was performed. Blood and tissue cultures grew methicillin-resistant Staphylococcus aureus. Combined medical and surgical treatment failed to achieve source control of the panfibular osteomyelitis despite multiple debridements. Given the life-threatening sepsis and the fact that the fibula was not salvageable due to the extent of the osteonecrosis, the patient underwent resection of the entire fibula. Muscles of his left leg anterior compartment were also debrided due to extensive myonecrosis. The patient remained in the ICU for a total of 27 days and received appropriate multidisciplinary care with infectious disease, orthopedics, and critical care.

Laboratory testing

Laboratory studies on initial presentation revealed a WBC of 36.6 K/uL, a neutrophil/lymphocyte ratio of 6.3, a blood urea nitrogen (BUN) of 16 mg/dL, and a C-reactive protein (CRP) of 18.2 mg/dL.

Imaging

**Figure 1:**
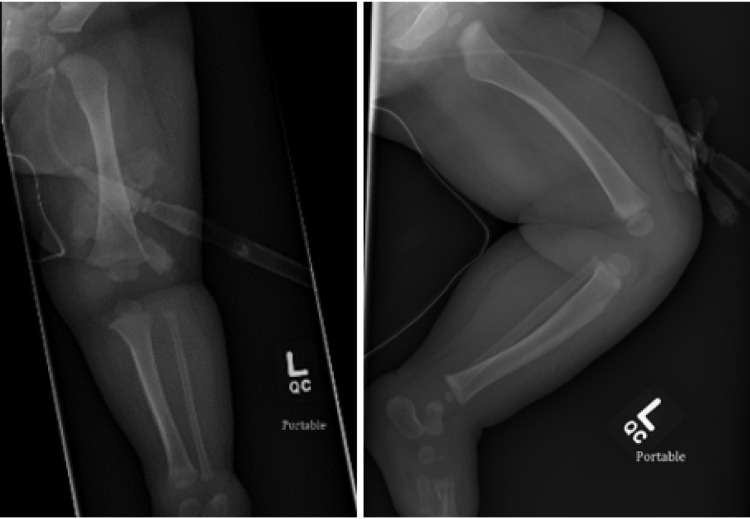
Radiography of the left leg AP (Pane A) and lateral (Pane B) of the left leg, before resection of the fibula, demonstrating extensive soft tissue edema.

Radiography of the lower limb revealed diffuse, right lower extremity soft-tissue edema (Figure 1).

**Figure 2:**
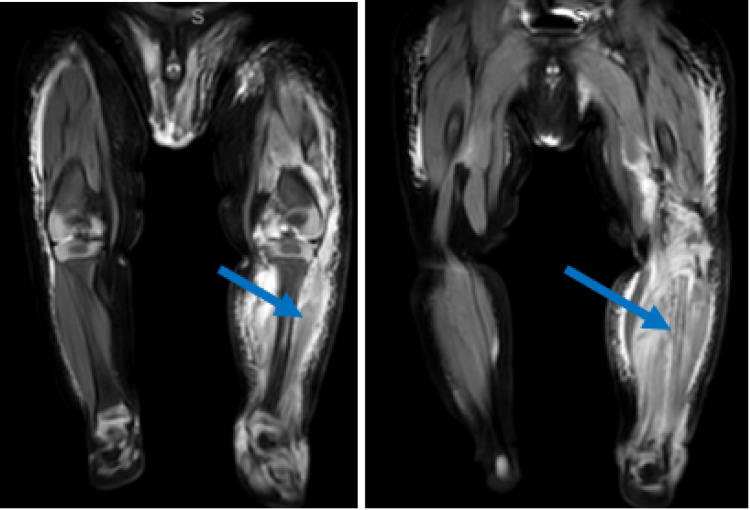
MRI of the left lower extremity T2 magnetic resonance imaging coronal images demonstrate an extensively abnormal signal in the left lower extremity, most markedly within the anterior lateral compartment, deep dorsal compartment (Panel A), and left fibula (Panel B), suggestive of myonecrosis and osteonecrosis.

T2-weighted MRI on initial presentation demonstrated extensively increased signal in the left lower extremity most markedly within the anterior lateral compartment and the deep dorsal compartment (Figure 2).

Differential diagnosis

The differential diagnosis for fibular osteomyelitis includes septic arthritis, tibial osteomyelitis, fracture, tubercular pseudotumor [12], primary neoplastic lesions, and metastases [2].

Complications

**Figure 3:**
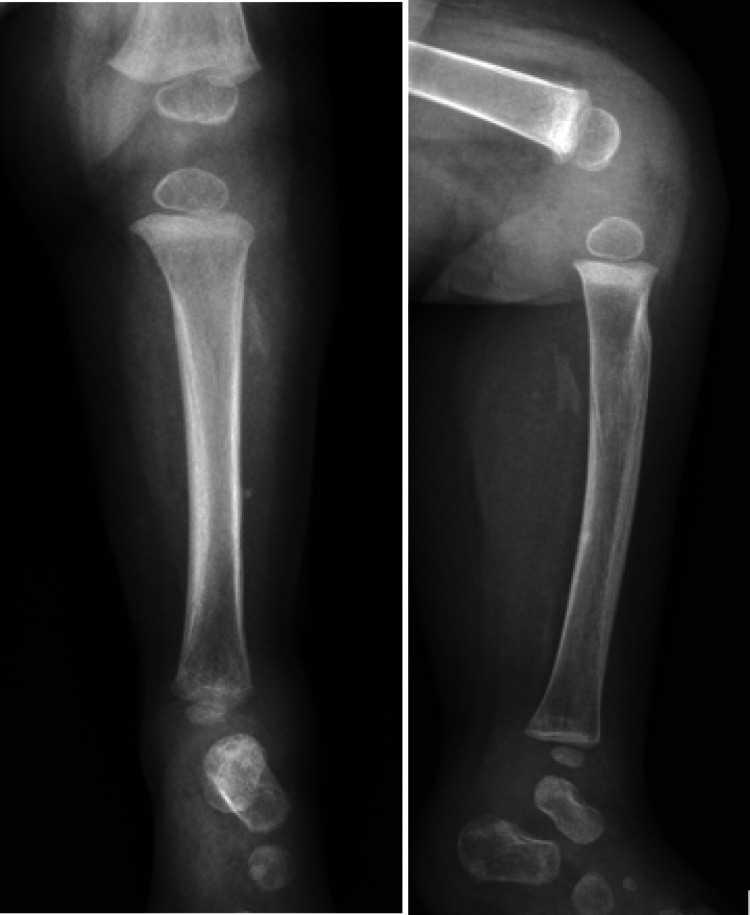
Radiography of the left leg after resection Anteroposterior (Panel A) and lateral (Panel B) of the left lower extremity after fibular resection.

The patient initially tolerated fibular resection well with no acute complications. Radiography demonstrated a stable interval of fibular resection (Figure 3).

**Figure 4:**
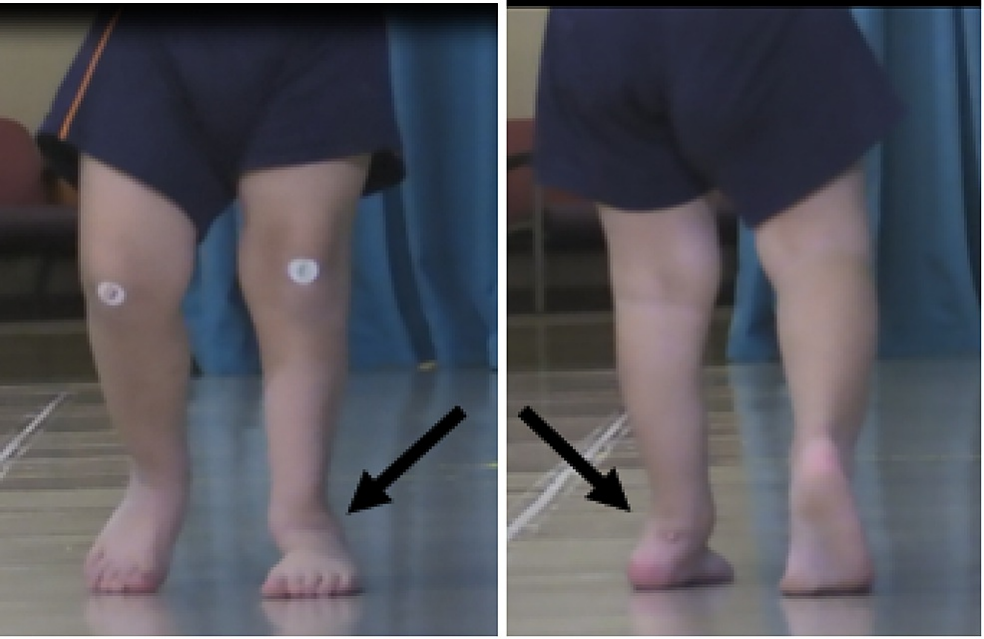
Two-dimensional clinical views Two-dimensional coronal view from the front (Panel A) and back (Panel B) demonstrating the valgus deformity with superolateral subluxation of the left ankle during late stance.

The patient subsequently developed an equinus deformity of the left ankle. Physical examination eight months after the fibular resection demonstrated a lack of ankle dorsiflexion, weakness of the anterior compartment muscles, and a fixed tendoachilles contracture. The patient also had a superolateral dislocation of the talus (Figure 4).

Treatment

Initially, we attempted to treat the patient conservatively with physical therapy and ankle-foot arthrosis (AFO). However, this did not slow the developing equinus. We thus proceeded with an open tendoachilles lengthening at 12 months after the fibular resection. However, in the subsequent months, the superolateral subluxation of the talus and progressive valgus deformity with brace intolerance worsened.

**Figure 5:**
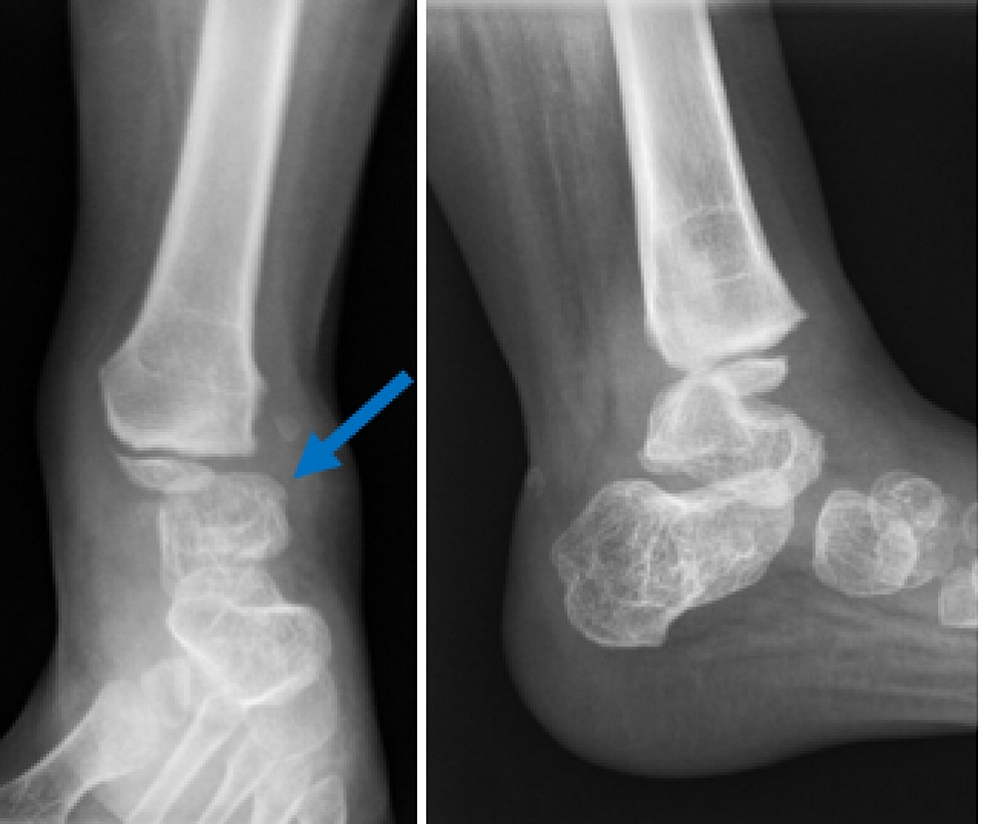
Radiography with deformity Anteroposterior (Panel A) and lateral (Panel B) views of the left ankle 21 months after fibular resection demonstrate superolateral subluxation of the talus with valgus deformity of the ankle.

Radiography demonstrated superolateral subluxation of the talus with valgus deformity (Figure 5).

**Figure 6:**
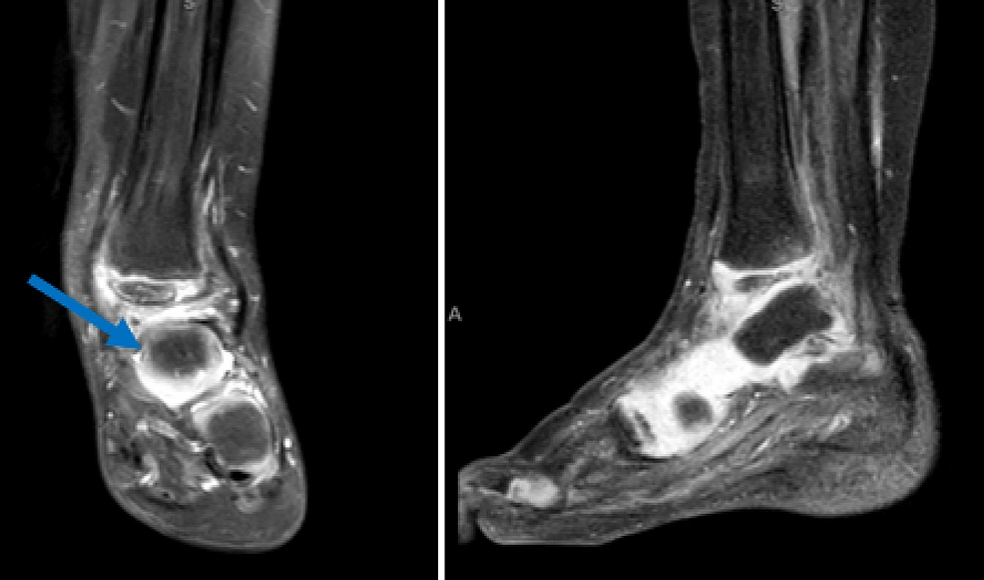
MRI demonstrating deformity A magnetic resonance imaging study of the left ankle demonstrates superolateral subluxation of the talus relative to the distal tibia in the coronal plane (Panel A) and sagittal plane (Panel B).

MRI of the left ankle 23 months after fibular resection demonstrated a complete tear of the deltoid ligament (Figure 6).

Given poor lower extremity alignment (Figure 4), progressive valgus deformity with superolateral subluxation of the talus (Figure 5 and Figure 6), difficulty with ambulation, and poor brace tolerance, surgical options were discussed with the family.

The surgical options of tibiotalar arthrodesis, lateral malleolarplasty, and a Syme amputation [13] were presented. The family favored the tibiotalar arthrodesis. The risk of a leg-length discrepancy, given the 3 mm/year contribution of the distal tibial growth plate toward limb length, was discussed. The patient had 13 remaining years until skeletal maturity and thus a final leg-length discrepancy of 3.9 cm was estimated. The potential need for a future leg-length equalization procedure was discussed. The family elected to proceed with the tibiotalar arthrodesis as the best option to provide long-term stability as well as improved brace tolerance.

**Figure 7:**
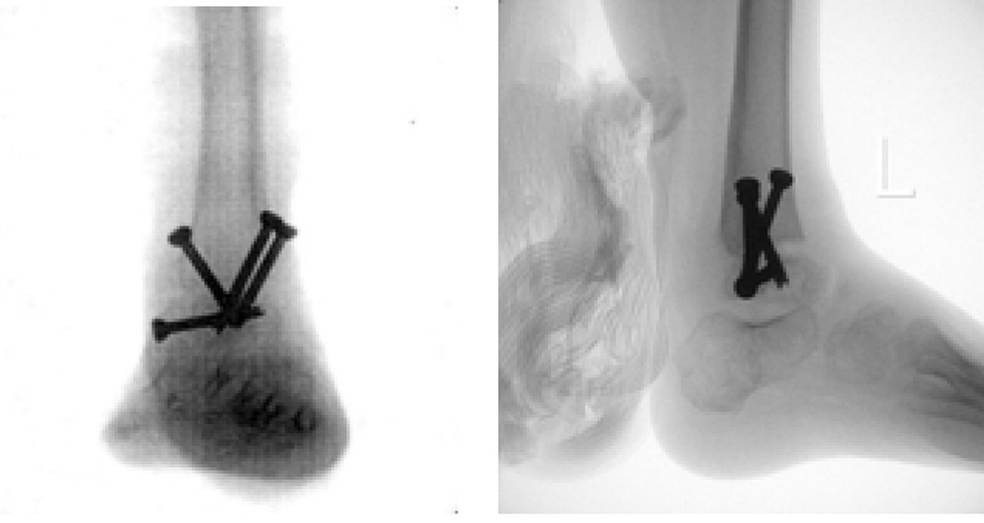
Intraoperative fluoroscopy Posteroanterior (Panel A) and lateral (Panel B) intraoperative radiograph demonstrating a left tibiotalar arthrodesis with internal fixation and resolved superolateral subluxation.

A left tibiotalar arthrodesis was performed 27 months after the fibular resection. An anteromedial approach was used and screw fixation across the joint was pursued. 4.0 cannulated cancellous screws were used to fix the ankle in neutral dorsiflexion, five degrees of valgus, and 10 degrees of external rotation (Figure 7).

He was placed in a long-leg non-weight-bearing cast postoperatively for six weeks.

Follow-up and outcome

At the six-week follow-up, the cast was discontinued, and he was sent to physical therapy for gait training and was allowed to weight bear fully with a walker on his left lower extremity. At the three-month follow-up, the ankle was in a plantigrade position, and he was able to ambulate well independently.

**Figure 8:**
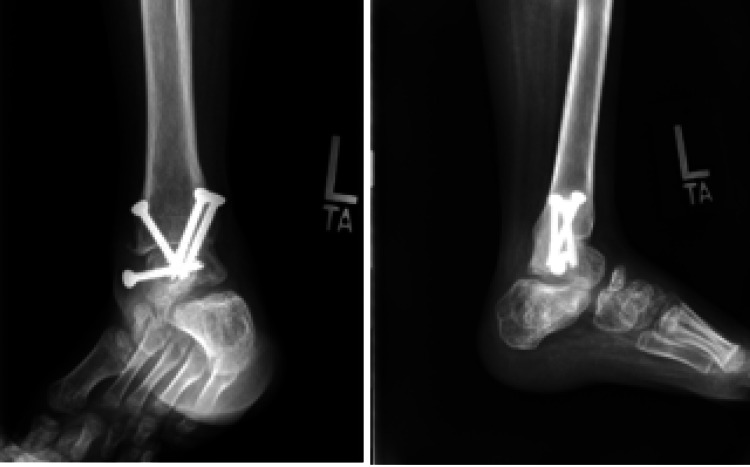
Follow-up radiography Anteroposterior (Panel A) and lateral (Panel B) radiographs of the left ankle 14 months after the procedure demonstrate a consolidated tibiotalar arthrodesis.

**Figure 9:**
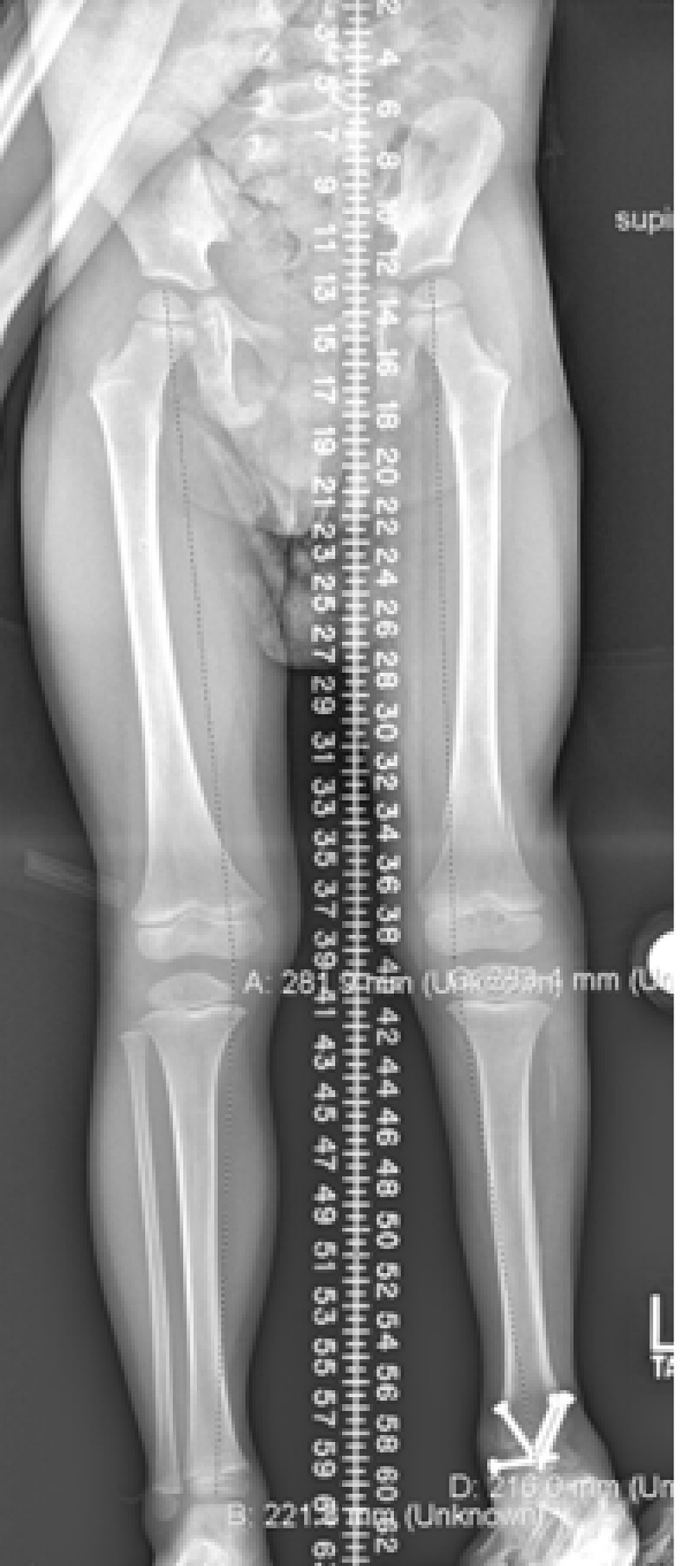
Follow-up bone length study A bone length study from 14 months after the arthrodesis demonstrates a minor leg-length discrepancy.

At the 14-month follow-up, the ankle remained in a plantigrade position and the patient was able to bear weight and ambulate with no pain. Radiographs demonstrated a completely fused tibiotalar joint (Figure 8) and a minor leg-length discrepancy of less than 1 cm (Figure 9).

The patient and family were counseled on the importance of follow-up until skeletal maturity.

## Discussion

Progressive subluxation of the ankle joint in the setting of fibular resection for panfibular osteomyelitis has not been reported previously. We report the reconstructive option for a case of progressive ankle subluxation after fibular resection for panfibular osteomyelitis.

The fibula plays an important role in both the static and dynamic stability of the lower extremity [14]. Load transmission through the fibula varies with ankle position; however, it averages 7% when the ankle is in the neutral position. Bozkurt et al. demonstrated that the proximal and distal fibulae are important in regard to knee and ankle stability, respectively [14]. Except for a mild secondary quadriceps weakness, middle fibula resection did not cause a significant biomechanical disturbance in gait [14].

Partial or complete resection of the fibula results in lower extremity instability [15]. Resection of the proximal fibula has been shown to have significant varus laxity at the [16] and maybe related to knee instability as well [14]. However, ankle instability and spiral diaphyseal fractures of the tibia have been observed in children undergoing more than 2 cm of resection [17] and so fibular resection should be preceded by the exhaustion of other treatment options as done in the present case.

Though anatomic reconstruction is the preferred treatment for progressive ankle instability refractory to conservative treatment [17], this is not possible in the absence of the fibula. Little literature exists regarding the options for progressive ankle instability in the setting of fibular resection. Jung et al. demonstrated successful fibular reconstruction for the treatment of valgus instability status post distal fibular resection for Ewing’s sarcoma. Lateral ligament reconstruction (contralateral biceps femoris tendon) in addition to lateral malleolus reconstruction (reversed proximal fibular graft) were performed one-month post resection of the distal fibula in this study [18]. Weber et al. described a lateral malleolarplasty involving the implantation of a triangular iliac crest autograft to the lateral distal tibia and gluteal fascia autograft to approximate the lateral ligaments in the setting of partial fibular aplasia [19]. However, the anatomic ligament reconstruction in the ankle in the setting of panfibular resection has not been described in the pediatric population. As a very last resort, the Syme amputation can be considered a surgical option. However, it should only be considered with both a fibular deficiency and a concurrent leg-length discrepancy of greater than 20 cm [13]. In the setting of progressive subluxation status post fibular resection, arthrodesis may be the appropriate surgical option. It is important to consider the potential risks of limb-length discrepancy when arthrodesis occurs before physeal closure. For these reasons, these patients need to be followed until skeletal maturity. 

## Conclusions

Except in the case of life-threatening sepsis, fibular resection should be avoided until the formation of a stable involucrum. Tibiotalar arthrodesis is a viable salvage option for the treatment of progressive valgus ankle subluxation in the setting of fibular resection.
